# Genetic Diversity Analysis and GWAS of Plant Height and Ear Height in Maize Inbred Lines from South-East China

**DOI:** 10.3390/plants14030481

**Published:** 2025-02-06

**Authors:** Changjin Wang, Wangfei He, Keyu Li, Yulin Yu, Xueshi Zhang, Shuo Yang, Yongfu Wang, Li Yu, Weidong Huang, Haibing Yu, Lei Chen, Xinxin Cheng

**Affiliations:** 1 College of Agriculture, Anhui Science and Technology University, Fengyang 233100, China; wangchj@ahstu.edu.cn (C.W.); wangfei_he@163.com (W.H.); likeyu012@163.com (K.L.); yjs2023223@ahstu.edu.cn (Y.Y.); 19956257739@163.com (X.Z.); 19855685231@163.com (S.Y.); wangyongfu@ahstu.edu.cn (Y.W.); yul@ahstu.edu.cn (L.Y.); huangwd@ahstu.edu.cn (W.H.); yuhb@ahstu.edu.cn (H.Y.); 2 Engineering Technology Institute of Maize Breeding in Anhui Province, Fengyang 233100, China

**Keywords:** maize, inbred germplasm, genetic diversity, genome-wide association studies, plant height, ear height, candidate genes

## Abstract

Maize is a critical crop for food, feed, and bioenergy worldwide. This study characterized the genetic diversity and population structure of 212 important inbred lines collected from the Southeast China breeding program using the Maize6H-60K single nucleotide polymorphism (SNP) array. To investigate the genetic architecture of plant height (PH) and ear height (EH), genome-wide association analysis (GWAS) was performed on this population in 2021 and 2022. Cluster analysis and population genetic structure analysis grouped the 212 maize inbred lines into 10 distinct categories. GWAS identified significant associations for PH, EH, and the EH/PH ratio. A total of 40 significant SNP (*p* < 8.55359 × 10^−7^) were detected, including nine associated with PH, with phenotypic variation explained (PVE) ranging from 3.42% to 25.92%. Additionally, 16 SNP were linked to EH, with PVE ranging from 2.49% to 38.49%, and 15 SNP were associated with the EH/PH ratio, showing PVE between 3.43% and 16.83%. Five stable SNP, identified across two or more environments, were further analyzed. Three of these SNP loci are reported for the first time in this study: two loci associated with the PH, AX-108020973, and AX-108022922, as well as one new locus, AX-108096437, which was significantly associated with the EH/PH ratio. Additionally, two other significant SNP (AX-247241325 and AX-108097244) were located within a 2 Mb range of previously identified QTL and/or related SNP. Within the 200 kb confidence intervals of these five stable SNP loci, 76 functionally annotated genes were identified. Further functional analysis indicated that 14 of these genes may play a role in regulating plant morphology, which is primarily involved in hormone synthesis, microtubule development, root growth, and cell division regulation. For instance, the homologous genes *GRMZM2G375249* and *GRMZM2G076029* in maize correspond to *OsPEX1* in rice, a protein similar to extension proteins that are implicated in lignin biosynthesis, plant growth promotion, and the negative regulation of root growth through gibberellin-mediated pathways. The candidate gene corresponding to AX-108097244 is *GRMZM2G464754*; previous studies have reported its involvement in regulating EH in maize. These findings enhance the understanding of QTL associated with maize plant-type traits and provide a foundation for cloning PH, EH-related genes. Therefore, the results also support the development of functional markers for target genes and the breeding of improved maize varieties.

## 1. Introduction

Maize (*Zea mays* L.) is the most important crop worldwide, serving as a primary source of food, feed, and bioenergy [1]. Its yield and quality are critical for ensuring a steady growth in national grain production, supporting the livestock industry, and improving the overall quality of life. Projections suggest that the average annual maize yield per unit area must double by 2050 to meet increasing demand [2]. In recent years, the sown and cultivated areas of maize have steadily expanded. Maize has progressively become a key food, feed, and cash crop in China and globally. Studies highlight that optimizing the density and compactness of maize varieties is crucial for increasing yield per unit area. Among the factors influencing density tolerance, PH plays a vital role. An optimal PH not only improves nutrient utilization but also enhances lodging resistance under high planting density [3]. Consequently, semi-dwarfing genetic improvement of maize PH has emerged as a major breeding objective [4].

Maize PH and EH are typical quantitative traits regulated by multiple genes. PH is one of the most important traits in maize yield research. Since PH is easily observable and its heritability exceeds 90% [5,6,7], breeders use it, along with EH, as a selection trait that indirectly reflects maize plant architecture during the breeding process. QTL mapping is an effective method for studying the genetic basis of complex crop traits. In recent years, advances in molecular biology have shifted the focus to genome-wide association analysis (GWAS), linkage genetic analysis, and transgenic technology. Additionally, continuous progress in high-throughput sequencing, the successful sequencing of various plant genomes, the development of numerous high-quality molecular markers (particularly single nucleotide polymorphisms), and the reduction in acquisition costs have provided a solid foundation for association analysis. Therefore, GWAS has become a key method for identifying genetic variations in complex crop traits and discovering target genes. In maize, the genetic analysis of plant type trait regulatory loci is conducted from two main perspectives. One approach is GWAS, while the other is linkage mapping analysis based on parental materials. Previous studies have successfully identified over 200 QTL sites related to maize PH across 10 linkage groups [8]. At present, over 30 dwarf genes have been cloned, most of which are involved in the synthesis, transport, and signal transduction pathways of plant hormones. The gibberellin pathway encompasses *An1*, *An2*, *Ct2*, *D1*, *D2*, *D3*, *D5*, *D8*, *D8-1023*, *D9*, *D11*, *GID1*, *Kn1*, and *Rd2* [9,10,11,12,13,14,15,16,17,18,19]. The auxin pathway comprises *Br2*, *Bv1*, *Te1*, *Vt2*, and *ZmPIN1a* [20,21,22,23]. The brassinolide pathway involves *Brd1*, *BRI1a*, *Lil1*, *Na1*, and *Na2* [24,25,26,27,28]. The ethylene biosynthesis gene is *Sdw3* [29], while the chrysololactone synthesis pathway gene is *ZmCCD8* [30]. Genes regulating microtubule skeleton arrangement include *Tan1* and *ZmRPH1* [31]. The photoperiod response gene is *ZmPHYC* [32], and the gene regulating meristem development is *D2003* [33]. Furthermore, Strigolactones (SLs) are a relatively newly identified class of plant hormones that are involved in the maintenance of plant architecture and exert critical control over lateral branching, such as the *LBO* homologs gene in maize [34]. From the above studies, the number of successfully cloned PH genes in maize remains limited, with most mutations leading to undesirable traits. For example, mutations in genes regulating hormone pathways can cause significant shortening of maize nodes, greatly reducing PH and limiting source supply capacity, which adversely affects maize yield. Therefore, there is an ongoing need to intensify efforts in gene mining for maize PH and EH traits, to deeply analyze their molecular regulatory mechanisms, and to identify novel PH regulation genes or beneficial alleles. These efforts could provide valuable references for maize molecular breeding.

While extensive investigations have been conducted on global maize germplasm through population structure analysis and GWAS for genetic locus identification, the distinct agroecological constraints in southeastern China, characterized by compound stresses including thermal extremes, pathogen pressures, and alternating drought–flood regimes, have shaped unique germplasm reservoirs. Notably, the genetic architecture of novel inbred lines derived from the bicyclic purification of hybrid cultivars in this ecoregion remains poorly characterized. To address this knowledge gap, we implemented a multidimensional analytical approach combining high-density SNP genotyping (Maize6H-60K array) with phenotypic evaluation across multi-environment trials. Our investigation of 212 region-specific inbred lines achieved three primary objectives: systematic germplasm characterization, the elucidation of critical genetic diversity patterns for breeding program design, and the identification of genomic regions governing PH and EH traits. Despite the many studies that have located and cloned QTL and genes associated with PH and EH, only a limited number of these loci have been effectively applied in maize-breeding practices. Moreover, due to the differences in materials and detection methods used in different studies, the identified QTL loci also vary. Therefore, further in-depth research is necessary to elucidate the genetic mechanisms underlying PH and EH in maize inbred lines specific to southeastern China. Our study establishes an association mapping framework integrating a carefully curated panel of 212 ecoregion-adapted inbred lines; multi-year, multi-location phenotypic datasets (PH, EH, EH/PH); and advanced GWAS protocols accounting for population stratification. Through this approach, we identified stable QTL and prioritized candidate genes showing consistent associations across environments. These findings provide both a theoretical foundation for the molecular dissection of plant architectural traits and practical markers for precision breeding in southeastern China’s maize improvement programs.

## 2. Results

### 2.1. Analysis of Plant-Type Traits and Phenotypes

**Figure 1:**
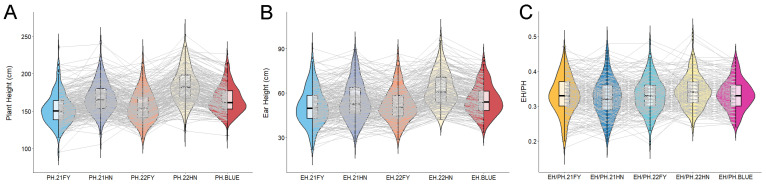
Phenotypic variation and distribution of plant height (**A**), ear height (**B**), EH/PH ratio (**C**) phenotypes of the 212 maize inbred lines. Violins and box plots depict the phenotypic distribution of the 212 maize inbred lines (four environments and the BLUE value of PH, EH, EH/PH ratio). FY refers to Fengyang County, Anhui Province, while HN denotes Sanya City, Hainan Province. The notation “PH.21FY” refers to the PH of the maize inbred lines cultivated in Fengyang, Anhui in 2021, whereas “PH.21HN” indicates the PH of the maize inbred lines grown in Sanya, Hainan in the same year. This naming convention applies similarly to other designations.

For the PH trait, the mean value ranged from 152.68 cm to 183.66 cm, with pheno-typic variation spanning 95.30 cm to 251.8 cm and a coefficient of variation between 12.34% and 13.81% (Table 1 and Table S1). For the EH trait, the mean value varied from 50.96 cm to 62.94 cm, phenotypic variation extended from 21.88 cm to 99.00 cm, and the coefficient of variation ranged from 18.77% to 24.12% (Table 1 and Table S1). Regarding the EH/PH ratio, the mean value ranged from 0.32 to 0.34 across the four environments, with phenotypic variation between 0.18 and 0.51 and a coefficient of variation from 13.40% to 16.45% (Table 1 and Table S1). The coefficient of variation for all three traits in each environment exceeded 10%, indicating abundant phenotypic variation in the investigated population. The data distribution for PH, EH, and the EH/PH ratio followed a normal distribution trend (Figure 1), suggesting that these traits conform to a quantitative inheritance model regulated by multiple genes.

### 2.2. Correlation Analysis of Plant-Type Traits

**Figure 2:**
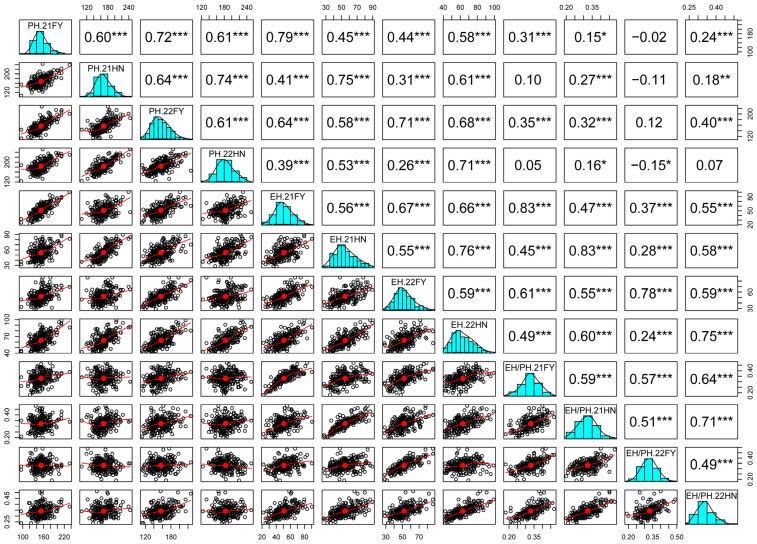
Correlation analyses between plant height (PH), ear height (EH), and the ratio of EH/PH (EH/PH) phenotypes of the 212 maize inbred lines. * indicates *p* < 0.05, ** indicates *p* < 0.01, *** indicates *p* < 0.001.

Correlation analysis revealed that the same trait across different environments was significantly correlated (*p* < 0.01). Analysis of plant-type traits in 212 maize inbred lines showed that the correlation coefficients for PH, EH, and the EH/PH ratio were 0.60–0.74, 0.55–0.76 and 0.49–0.71, respectively (Figure 2). Further analysis of different phenotypic traits demonstrated a significant positive correlation between PH and EH (*p* < 0.01), with correlation coefficients from 0.26 to 0.79. EH and the EH/PH ratio also exhibited a significant positive correlation (*p* < 0.01), with correlation coefficients between 0.24 and 0.83. Meanwhile, PH and the EH/PH ratio were significantly correlated (*p* < 0.01), with coefficients ranging from −0.15 to 0.41. The generalized heritability values for PH, EH, and the EH/PH ratio were 0.81, 0.81, and 0.80 (Table 1), respectively, indicating that these traits are predominantly influenced by genetic factors and warrant further analysis.

### 2.3. Population Structure and Kinship Analysis

**Figure 3:**
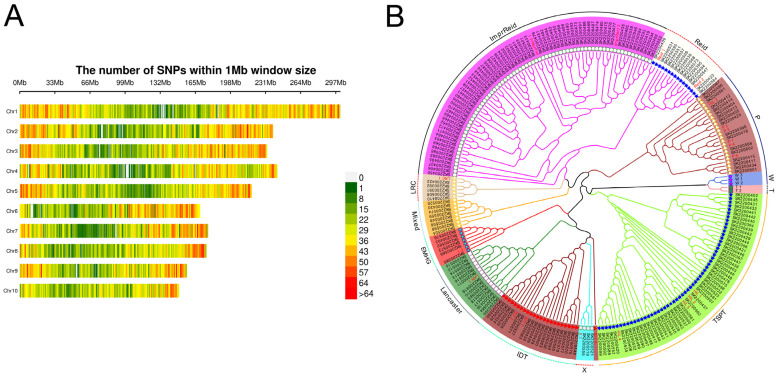
The density distribution of SNP on chromosome (**A**) and Phylogenetic tree of 212 maize inbred lines (**B**). EMHG (Early-maturing hard-grain cluster); IDT (Iodent group); ImprReid (Improved Reid group); Lancaster (Lancaster group); LRC (Lvda Red bone group); Mixed (Mixed group); P (P group); Reid (Reid group); TSPT (Tang Si Ping Tou group); X (X group); W (Local glutinous group); T (Tropical group). The various colored lines denote distinct maize germplasm groups.

After quality control, the remaining 58,445 high-quality SNPs were distributed across 10 chromosomes. Specifically, the number of SNPs on chromosomes 1 to 10 was 9552, 7344, 7264, 6591, 6423, 5068, 4991, 4886, 4873, and 4232, respectively. At the genome-wide level, the distribution of SNPs per 1 Mb window suggests a relatively high SNP density at the ends of each chromosome and a lower density in the middle regions (Figure 3A). Cluster analysis was performed using 26 known types of maize representative inbred line materials and the 212 materials in this study (Table S2). The results classified the experimental materials into 10 groups: Improved Reid group (ImprReid, 71 lines); P group (P, 17 lines); Tang Si Ping Tou group (TSPT, 53 lines); Iodent group (IDT, 21 lines); Lancaster group (Lancaster, 14 lines); Early-maturing hard-grain cluster (EMHG, 6 lines); Lvda Red bone group (LRC, 5 lines); Reid group (Reid, 14 lines); X group (X, 3 lines); Mixed group (Mixed, 8 lines). (Figure 3B, Table S2).

**Figure 4:**
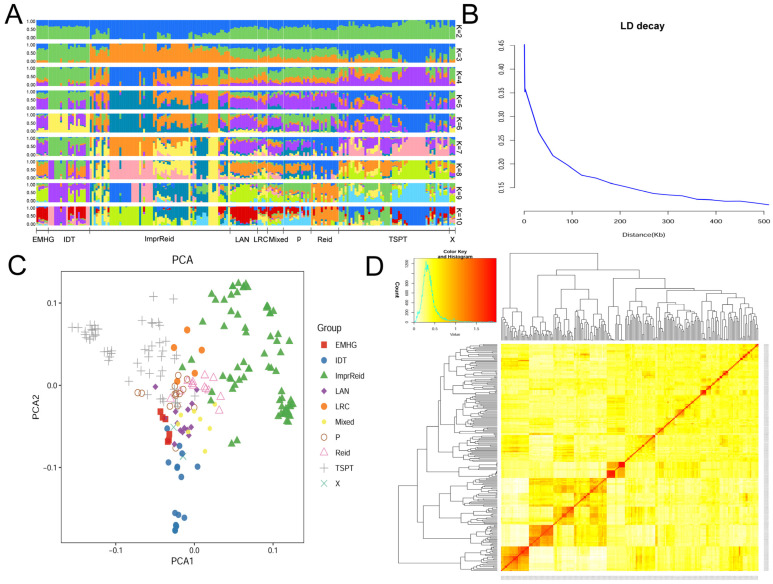
Genetic diversity and population structure analysis of 212 maize inbred lines. (**A**) Population structure of 212 maize inbred lines. EMHG (Early-maturing hard-grain cluster); IDT (Iodent group); ImprReid (Improved Reid group); LAN (Lancaster group); LRC (Lvda Red bone group); Mixed (Mixed group); P (P group); Reid (Reid group); TSPT (Tang Si Ping Tou group); X (X group). (**B**) Linkage disequilibrium (LD) decay of 212 maize inbred lines (r^2^ > 0.15). (**C**) Principal component analysis of 212 maize inbred lines. The different groups represented by different colors, and scattered points with the same color are basically clustered together. (**D**) Kinship heatmap of 212 maize inbred lines.

PLINK1.9 and ADMIXTURE1.3 software were used to construct the population structure. Based on the cross-validation error rate, the optimal K value, and the clustering of typical inbred lines, the inbred lines were divided into 10 groups (Figure 4A). Linkage disequilibrium (LD) analysis revealed an LD attenuation distance of approximately 200 kb when R^2^ = 0.15, which was used for candidate gene prediction analysis in the GWAS (Figure 4B). Principal component analysis (PCA) further categorized the 212 materials into 10 classes (Figure 4C). According to the affinity heat map (Figure 4D), the affinity distance between materials within each group was evident. Darker colors indicated higher affinity coefficients, signifying greater similarity and a higher likelihood of belonging to the same subgroup. The relationship coefficients were mainly concentrated within the range of 0.3 to 0.5, reflecting a relatively simple relationship structure. Most inbred lines in the population appeared to be either unrelated or outbred lines.

### 2.4. Genome-Wide Association Analysis

**Figure 5:**
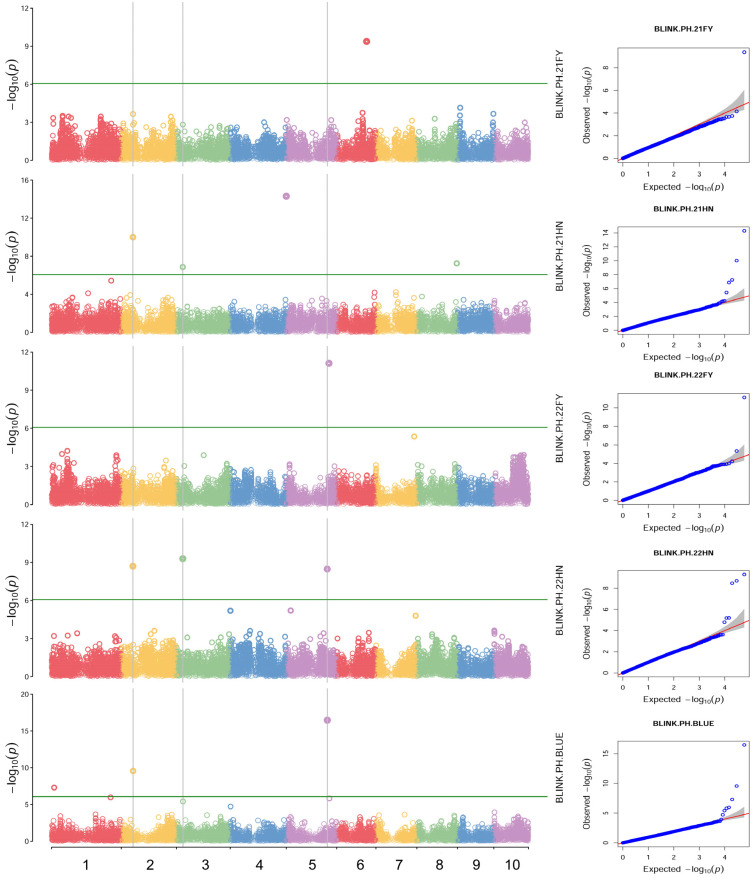
Manhattan map (**left**) and QQ plots (**right**) of plant height (PH) in four environments and the BLUE value. Each circles on the left figures represents an SNP, and the green lines represents the threshold of <8.55359 × 10^−7^. Different colors represent different chromosomes. The red lines on the right figures are the trend lines to which the ideal QQ plot in each case should correspond.

To account for population structure and principal components, the ‘BLINK’ model was used to perform a genome-wide association analysis of PH, EH, EH/PH ratio, and BLUE values across four distinct environments. SNP markers from maize natural populations sequenced using the Maize6H-60K chip were employed. Comprehensive whole-genome association analyses were conducted using a threshold value of *p* < 8.55359 × 10^−7^ (−lg*p* > 6.07) to evaluate PH, EH, and the EH/PH ratio. A total of 40 SNP significantly associated with these traits were identified on chromosomes 1 through 10 (Figure 5). Specifically, nine SNP sites were significantly associated with PH, with phenotypic variation explained by individual SNPs ranging from 3.42% to 25.92%. Sixteen SNP loci were significantly associated with EH, explaining 2.49% to 38.49% of the variation. Additionally, 14 SNP loci were significantly associated with the EH/PH ratio, with explanatory power ranging from 3.43% to 16.83%.

Stable SNP loci, defined as those detected in two or more environments for each trait, were identified for PH, EH, and the EH/PH ratio. For PH, three stable loci were found on chromosomes 2, 3, and 5 across the four environments analyzed (Figure 5). The SNP AX-107984795 had the lowest *p*-value (5.02 × 10^−15^), while AX-86272584 demonstrated the highest percentage of variance explained (PVE) of 25.92%.

**Figure 6:**
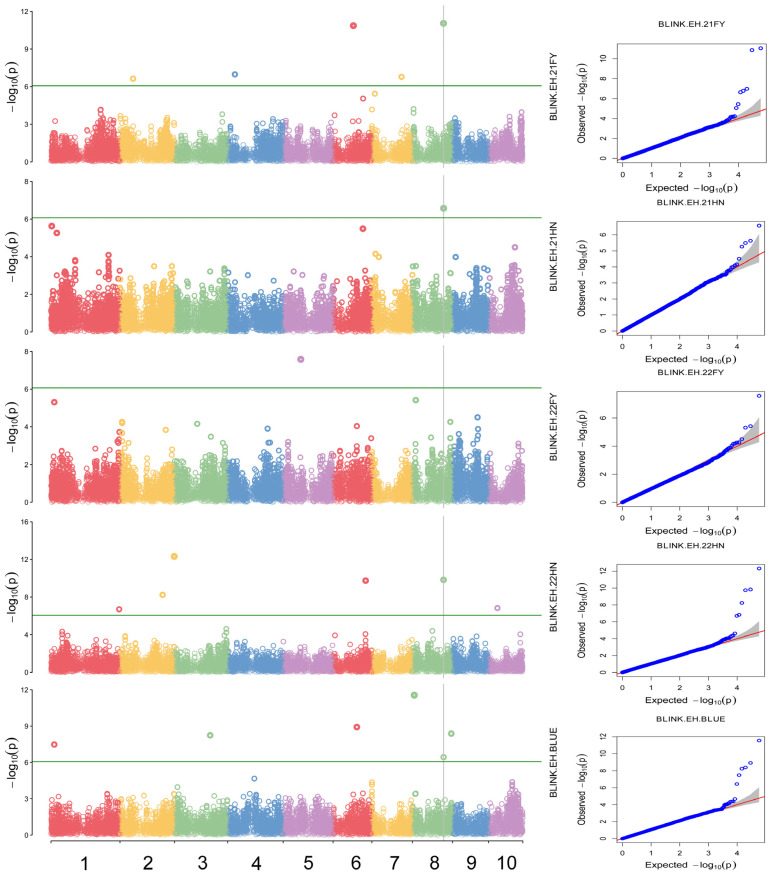
Manhattan map (**left**) and QQ plots (**right**) of ear height (EH) in four environments and the BLUE value. Each circles on the left figures represents an SNP, and the green lines represents the threshold of <8.55359 × 10^−7^. Different colors represent different chromosomes. The red lines on the right figures are the trend lines to which the ideal QQ plot in each case should correspond.

Notably, for EH, a significant SNP on chromosome 8 (AX-108097244) exhibited *p*-values ranging from 8.94 × 10^−12^ to 3.63 × 10^−7^, explaining 5.02% to 38.49% of phenotypic variation (Table 2), and was consistently detected across all four environments (Figure 6).

**Figure 7:**
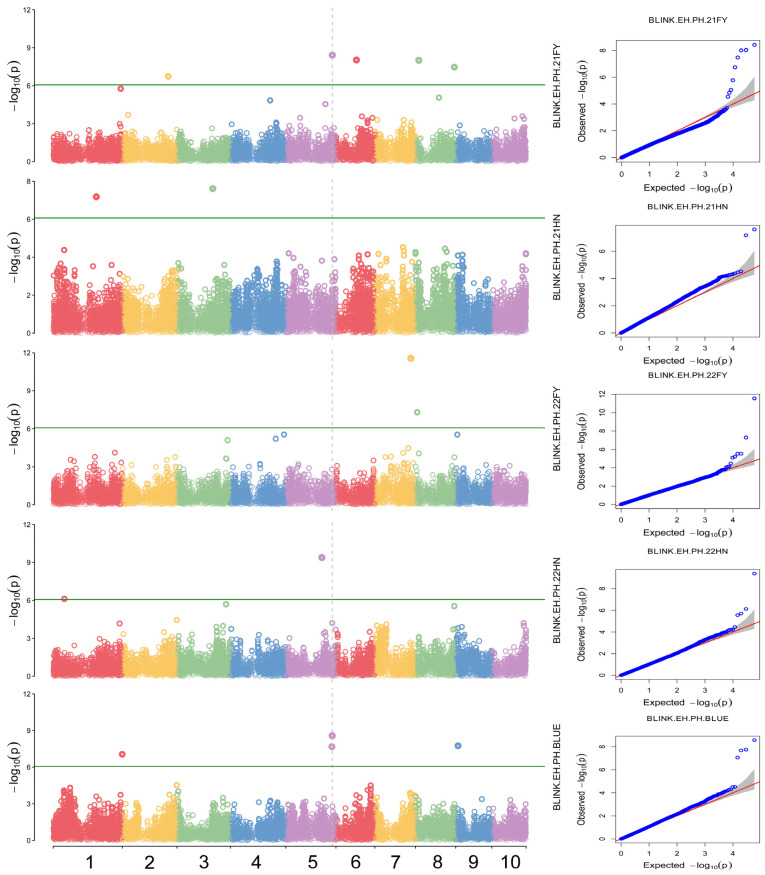
Manhattan map (**left**) and QQ plots (**right**) of the EH/PH ratio in four environments and the BLUE value. Each circles on the left figures represents an SNP, and the green lines represents the threshold of <8.55359 × 10^−7^. Different colors represent different chromosomes. The red lines on the right figures are the trend lines to which the ideal QQ plot in each case should correspond.

Additionally, a stable SNP site in both environmental conditions for the EH/PH ratio (AX-108096437) was detected on chromosome 5 (Figure 7), with a *p*-value of 2.69 × 10^−9^ and a high PVE of 6.23% (Table 2).

### 2.5. Candidate Gene Analysis

Three characters yielded five stable SNP loci when detected against the B73 RefGen_v3 reference genome (https://www.maizegdb.org/ accessed on 20 May 2024). Candidate gene identification within a ±200 Kb range upstream and downstream of each locus revealed a total of seventy-six candidate genes. Of these, thirty-one have functional annotations listed in Table S3. Further investigation identified fourteen genes potentially involved in plant growth and development processes.

For specific traits related to PH characteristics, three primary-effect loci containing eight candidate genes were identified. These include *GRMZM2G113629*, which encodes protein disulfide isomerase-like proteins; *GRMZM2G375249*, an extensin-like protein; *GRMZM2G076029*, a leucine-rich repeat extensin-like protein; *GRMZM2G152689*, a phosphatidylethanolamine-binding protein; *GRMZM2G099989*, an exocyst complex component SEC6; *GRMZM2G316366* and *GRMZM2G492156*, MADS box transcription factors that are highly expressed in roots; *AC210598.3_FG003*, a phytochelatin synthase PCS1.

One major locus associated with EH contained three candidate genes, WD-40 repeat family proteins encoded by *GRMZM2G464754*, DCD domain proteins encoded by *AC207342.3_FG008*, and ubiquitin-specific protease 7 represented by gene ID *GRMZM2G028733*.

Within a major effect locus linked to the EH/PH ratio, three distinct candidates were identified. These include *GRMZMZG147377*, which contributes to lateral stability in cor-tical microtubules alongside *CMU1* and *CMU22*, and two other genes: *GRMZMZG125432*, a sequence-specific DNA-binding transcription factor, and *GRMZMG170595*, encoding histidinol phosphate aminotransferase.

### 2.6. Effect Analysis of Allelic Variation

The dominant allele, which exhibited the highest percentage variance explained among stable SNPs associated with attributes such as PH, EH, and the EH/PH ratio, was selected for further evaluation to investigate the effects of variations within these alleles.

**Figure 8:**
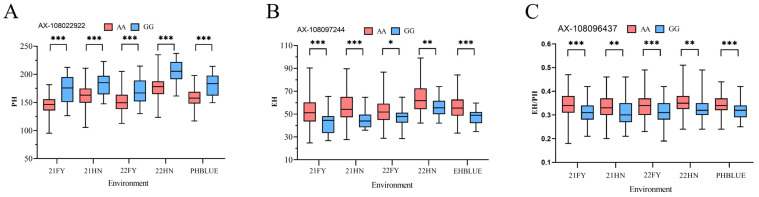
Allelic effects of the SNP associated with plant height (PH), ear height (EH), and the ratio of EH/PH (EH/PH) traits. (**A**) The allelic effect of AX-108022922 locus on PH. (**B**) The allelic effect of AX-108097244 locus on EH. (**C**) The allelic effect of AX-108096437 locus on EH/PH ratio. * indicates *p* < 0.05, ** indicates *p* < 0.01, *** indicates *p* < 0.001.

For PH, differing alleles at location AX-108022922 resulted in notable differences ob-served across various trials conducted in the years ‘21FY’, ‘21HN’, ‘22FY’, ‘22HN’, and PHBLUE. Average increases of 25.00 cm, 20.36 cm, 18.15 cm, 26.40 cm, and 22.66 cm, re-spectively, were recorded when comparing A/A versus G/G allele types (Figure 8A). For EH, comparisons between A/A and G/G variants at location AX-108097244 revealed average increments of 9.86 cm, 11.21 cm, 4.91 cm, 8.07 cm, and 8.54 cm, respectively. These differences were statistically significant and are visually represented in Figure 8B. Regarding the EH/PH ratio, the A/A allelic variation at AX-108096437 showed an average increase of 0.03 cm, 0.02 cm, 0.03 cm, 0.02 cm, and 0.02 cm compared to the G/G allelic variation (Figure 8C).

## 3. Discussion

### 3.1. The Significance of PH and EH in Maize

The breeding of “semi-dwarf” crops has initiated a green revolution in agriculture, significantly enhancing global grain production. An optimal plant height can effectively reduce lodging, improve tolerance to plant density, and enhance mechanization efficiency, thereby greatly promoting maize production. Maize plant architecture traits not only influence growth and development but also play a key role in determining maize yield and lodging resistance. PH and EH are significant components of maize plant architecture. GWAS is an essential technique for studying quantitative traits, analyzing the genetic structure of complex traits, and identifying genes. It is also a widely used method in the study of quantitative inheritance. Further exploration of functional SNP sites that affect PH and EH, along with functional verification of candidate genes, can provide valuable insights into the inheritance of phenotypic traits in maize plant architecture. In this study, GWAS was conducted using 212 maize inbred lines with a rich genetic background (Figure 3 and Figure 4). A total of five SNP markers were identified across multiple environments, supported by high heritability data from two years and four replicates (Table 1). These findings align with the results of Pan et al. (2017) [7] and Wang et al. (2018) [6]. Specifically, AX-108020973 was detected in both 22HN and BLUE; AX-108022922 was found in 21HN and 22HN; AX-247241325 was observed in 22HN and BLUE; AX-108097244 was present in 21FY, 21HN, 22HN, and BLUE; and AX-108096437 was identified in 21FY and BLUE. The observed high heritability of PH and EH creates favorable conditions for molecular marker-assisted selection breeding in maize. Analysis of the effects of colocalizing SNP alleles showed that different alleles of the colocalizing SNP detected in this study resulted in significant differences in traits and phenotypes (Table 2). This indicates the accuracy of the findings and the potential significance of the colocalizing SNP in developing molecular markers that regulate PH and EH.

### 3.2. Analysis of the Stable QTL in the Population

To date, approximately 30 dwarfing genes have been cloned in maize. However, the most extensively studied and utilized gene for dwarf breeding in maize remains *Brachytic2* (*Br2*). This prevalence is likely attributable to two primary factors: first, most identified dwarfing genes may be associated with linked loci that exert negative pleiotropic effects; second, different maize germplasms exhibit varying requirements for the degree of plant height improvement, as PH is a trait characterized by strong heterosis. Notably, Zhao et al. (2025) reported an innovative strategy that achieves universal and continuous reduction in PH through selective gene editing of the maize *Br2* gene. This approach provides significant genetic resources and technical support for enhancing maize phenotypes with improved density tolerance and resistance to lodging [35]. This study primarily focuses on two key areas. Firstly, 212 maize inbred lines purified from breeding programs in southeastern China were categorized through genotyping analysis, providing guidance for future breeding efforts. Secondly, recognizing the significance of maize plant architecture traits, this study conducted a GWAS on PH- and EH-related traits. The analysis identified five stable SNP that were consistently co-located across multiple environments, three of which are novel findings of this study, while two were located within the 2 Mb range of previously identified QTL or/and associated SNP.

In this study, a total of 40 significant SNP were associated with plant architecture traits. However, most QTL were detected only in a single environment, suggesting that PH and EH were significantly influenced by environmental factors. Among these, only three SNP markers on chromosomes 2, 3, and 5 were consistently detected for PH (Figure 5). Compared to previous studies, Peiffer et al. (2014) identified an SNP site regulating PH, located at 175,615,577 bp on chromosome 5 (using B73RefGen_v2 as the reference genome). This site is similar to the SNP (AX-247241325) found in this study on chromosome 5, with a difference of 1.62 Mb [5]. Additionally, the SNP (AX-247241325) found at 177,277,477 bp on chromosome 5 in this study falls within the common interval of the *qPH5-1* QTL reported by Zhou et al. (2018), located between 173.10 and 178.60 Mb on chromosome 5 [36]. For EH, 16 SNP markers were identified, with only the AX-108097244 SNP marker on chromosome 8 (located at 135948728 Mb) being consistently detected (Figure 6). The regulatory EH trait reported by Wang et al. [6] was located at 135.82 Mb on chromosome 8, with a difference of just 0.13 Mb. Due to limited studies on panicle position coefficient traits, no colocalized regions were found. In summary, of the five stable SNP loci identified in this study, two loci were consistent with previous localization studies, confirming the reliability of the association results. The identified SNPs provide a foundation for molecular marker-assisted selection breeding.

In this study, three stable SNPs were discovered for the first time, including two associated with PH, and among which AX-108020973 and AX-108022922 were detected in both environments (Figure 5). The PVE of these loci exceeded 5% and were dominant SNP (Table 2). Another new SNP locus, associated with EH/PH ratio, showed significant associations with AX-108096437, which could explain 5.58% of the phenotypic variation and was classified as a dominant SNP (Figure 7). These newly identified loci related to PH and EH/PH ratio are simultaneously applicable across different environments with high confidence, making them valuable for further analysis of the genetic structure of these traits.

Generally, PH in maize is positively correlated with yield. However, single-gene dwarf mutants often exert a significant negative impact on yield, making it challenging to achieve satisfactory applications in practical breeding programs. Therefore, it is advisable to focus research efforts on major QTL colocalized across multiple populations or to employ the strategy of pyramiding multiple PH QTL. This approach can facilitate the development of maize dwarf varieties with comprehensive desirable traits, including high density tolerance, appropriate growth duration, superior quality, strong stress resistance and stability, and high yield. Our analysis confirms the reliability of the experimental results. Compared to previous studies, the three stable SNP loci identified here do not share colocalization intervals, indicating that these loci may represent new QTL for maize plant type traits. Therefore, this study contributes to a deeper understanding of the QTL related to maize plant type traits.

### 3.3. A Comprehensive Analysis of Candidate Genes Prediction of the Stable QTL Interval in Maize

Several regulatory genes associated with PH and EH have been cloned, mainly including hormone-related genes such as auxin, gibberellin (GA), brassinolide (BR), and strigolactone (SL). Additionally, processes such as microtubule development, glucose metabolism, cellulose synthesis, root hair growth, and transcription factor regulation can influence plant stem development [37].

In this study, three stable loci associated with PH were identified. Among them, AX-108020973 contained the possible candidate gene *GRMZM2G113629*, which encodes PDI8, a type I endoplasmic reticulum transmembrane protein, and thiol-disulfide oxidase (Table 2). This work lays the foundation for future studies to identify the redox-regulated substrates of PDI8 and to clarify its distinct roles in cotyledon guard cells, newly expanding leaves, and the vasculature of plants [38]. AX-108022922 contains three potential candidate genes (Table 2). The homologs of *GRMZM2G375249* and *GRMZM2G076029* in rice are *OsPEX1*, an extensin-like protein involved in lignin biosynthesis, plant growth, and the negative regulation of root growth in a gibberellin-mediated manner [39]. The *Arabidopsis thaliana* homolog of *GRMZM2G152689* is *AT1G18100*, which encodes the phosphatidyl-ethanolamine-binding protein FT, a member of the *TFL1* family that responds to ABA signaling [40]. AX-247241325 includes four potential candidate genes: *GRMZM2G099989*, *AT1G71820*, which regulates cytokinesis via the exocyst subunit SEC6, and KEULE in *Arabidopsis thaliana* [41]. The genes *GRMZM2G316366* and *GRMZM2G492156* are classified as MADS-box transcription factors, with the former showing high expression in root tissues [42]. A report on the *Arabidopsis* homolog *AT5G44070* of *AC210598.3_FG003* indicates that growth is inhibited under cadmium exposure, resulting in progressive leaf chlorosis and the formation of a distinct brown pigment in roots [43].

For the EH, AX-108097244 includes three potential candidate genes (Table 2). Among these, *GRMZM2G464754* corresponds to *Zm00001d011140* in the B73 RefGen_v4 reference genome. Mutants lacking functional *Zm00001d011140* showed fewer male ear branches and reduced EH compared to wild-type plants [3]. The *AC207342.3_FG008* gene is involved in cellular and metabolic processes as well as biological regulation and signaling. WU et al. found that this gene primarily functions in ABA signaling pathways in *Arabidopsis thaliana* [44]. Additionally, the *GRMZM2G028733* gene encodes a ubiquitin-specific protease. Zhou et al. (2017) showed that this protease plays a crucial role in plant growth, development, and stress responses by primarily participating in protein deubiquitination processes [45]. Moon et al. (2009) further noted that deubiquitination can cause functional loss during rice growth, impacting overall development [46]. Thus, it is hypothesized that the candidate gene *GRMZM2G028733* may regulate plant growth and development through its involvement with ubiquitin-specific proteases.

For the EH/PH ratio, the AX-107996728 locus includes three potential candidate genes (Table 2). The homologous gene *AT3G27960* (*CMU2*) in *Arabidopsis thaliana* (*GRMZM2G147377*) is involved in cortical microtubule development and interacts with *CMU1* and the *Arabidopsis FRA1* kinase. This gene plays a crucial role in the growth and propagation of *Arabidopsis* [47]. The homologous gene *AT2G23740* (*SUVR5*) in *Arabidopsis* (*GRMZM2G125432*) exhibits delayed flowering and reduced root growth when mutated [48]. Additionally, the homologous gene for *GRMZM2G170595* in *Arabidopsis thaliana* is *AT5G10330* (*HPA1*). Mutations in this gene reduce free histidine content, impair root meristem function, and lead to significant shortening of the root system [49].

## 4. Materials and Methods

### 4.1. Plant Materials

This experiment used 212 diverse inbred maize lines provided by the Maize Breeding Engineering Technology Research Center of Anhui Science and Technology University (Table S2). The population was cultivated in four environments: Fengyang County, Anhui Province (FY, 32° N, 117° E), China, and Sanya City, Hainan Province (HN, 18° N, 109° E), China, during 2021 and 2022. These environments were designated as 21FY, 21HN, 22FY, and 22HN. The inbred lines were planted in single rows, each 5.0 m long, with a row-spacing of 0.6 m and a plant-spacing of 0.33 m, accommodating 15 plants per row. All other management practices followed conventional field protocols. Genetic data for 212 inbred lines and 26 representative inbred lines of various types used in the clustering analysis were provided by the Maize Research Center of the Beijing Academy of Agriculture and Forestry Sciences, Beijing, China (Table S2). Climate data for the maize cultivation periods in 2021 and 2022 are provided for Fengyang and Sanya in Supplementary Table S4.

### 4.2. Field Experiment and Phenotypic Measurements

At maturity, five individual plants were randomly selected from each family to measure PH and EH. Plant height is defined as the distance from the ground to the apex of the male spike, while ear height refers to the distance from the ground to the growing node of the first fruit spike; both measurements are expressed in centimeters (cm). The ear posi-tion coefficient (EH/PH) is the ratio of EH to PH [6]. Descriptive statistical analyses, in-cluding mean, standard deviation, coefficient of variation, range, skewness, and kurtosis, were conducted using IBM SPSS Statistics version 23.0 software on these data sets [50]. Additionally, Pearson correlation coefficient analysis and normal distribution tests were performed. To calculate generalized heritability for traits related to plant height and ear position, a linear mixed model was employed using the lme4 software package within R language, following this formula:

$$H^{2}=\sigma_{g}^{2}/\left(\sigma_{g}^{2}+\sigma_{ge}^{2}/n+\sigma_{e}^{2}/nr\right)$$

In the formula, $\sigma_{g}^{2}$ denotes genetic variation, $\sigma_{ge}^{2}$ represents the genotype-environment interaction, $\sigma_{e}^{2}$ indicates residual error, *n* refers to the number of environments, and *r* signifies the number of repetitions within each environment [51]. To account for the impact of planting in different years and regions on phenotypic traits, the lme4 package [52] was used to calculate the best linear unbiased estimations (BLUE) for PH, EH, and EH/PH ratio across various regions over multiple years. These BLUE values were then used as phenotypes for further GWAS.

### 4.3. DNA Extraction and Genotype Detection

Leaf samples were collected from related plants in the natural population when maize reached the five-leaf stage. DNA extraction followed the CTAB method [53]. Genotyping was performed using the whole-genome SNP chip Maize6H-60K developed by the Maize Research Center at the Beijing Academy of Agriculture and Forestry Sciences [54]. During genotyping, SNPs with a heterozygosity rate > 10%, an allele frequency < 5%, or a deletion rate > 20% were excluded. This process yielded a total of 58,455 SNP markers.

### 4.4. Population Structure and Linkage Disequilibrium (LD) Analysis

The subgroup structures of 212 maize materials were investigated using the ADMIXTURE Version 1.3 model-based clustering algorithm [55]. The PopLDdecay3.40 software was used to assess relationships among materials within populations and to calculate linkage disequilibrium coefficients (r^2^). Decay diagrams illustrating linkage disequilibrium (LD) were also generated. Phylogenetic trees were constructed based on adjacency methods using the Neighbor-Joining method in MEGA7 software, with visualizations provided through “Evolview v2” available at [56].

### 4.5. Genome-Wide Association Study

The “BLINK” model from the R package was used to perform a genome-wide association analysis of PH, EH, and the EH/PH ratio [57,58]. Population structure and a principal components analysis (PCA) were included as covariates. The threshold for GWAS was set at −log(p) = 6.07 based on Bonferroni correction. Significant correlations were evaluated using Q-Q plots and *p*-value distributions. Candidate genes were predicted based on the linkage disequilibrium (LD) decay distance, which for this population was determined to be 200 kb (r^2^ = 0.15). The MaizeGDB database, referencing the B73 RefGen_v3 genome, served as a reference, as this genomic version was used for both SNP chip data design and subsequent transcriptome data calculations.

### 4.6. Functional Annotation of Candidate Genes

In this study, candidate genes for SNP loci consistently detected across association analyses were identified. Based on LD structural analysis, candidate genes were defined as those located within 200 kb upstream or downstream of the markers. Relevant biological information about these genes was gathered using the MaizeGDB database (https://maizegdb.org/gbrowse/maize_v3/, accessed on 20 May 2024), along with NCBI (https://www.ncbi.nlm.nih.gov/ accessed on 22 May 2024) gene databases and UniProt (https://www.uniprot.org/ accessed on 20 June 2024) gene databases as references. Functional mining based on this information was conducted to predict potential candidate genes.

## 5. Conclusions

A total of 212 inbred lines were used as associated populations, with 58,445 SNP markers employed. Population structure analysis successfully classified the samples into 10 groups, revealing the diversity of maize germplasm resources and the distribution of genetic variation. The BLINK model was then used for genome-wide association analysis of PH, EH, and the EH/PH ratio, identifying 40 significant associated SNPs. Among these, five stable SNPs were detected across two or more environments, three of which were identified for the first time in this study. During the analysis of the 200-kb confidence intervals surrounding five stable SNP loci, a total of 76 candidate genes with functional annotations were identified. Further functional analysis indicated that 14 of these genes are potentially involved in regulating plant architecture. For example, the homologs of *GRMZM2G375249* and *GRMZM2G076029* in rice, *OsPEX1*, encode an extensin-like protein. This protein plays a role in lignin biosynthesis and plant growth, and it negatively regulates root growth through a gibberellin-mediated pathway. Moreover, the candidate gene at locus AX-108097244, *GRMZM2G464754*, has been shown in previous studies to influence EH in maize. These findings contribute to research on maize plant type traits and provide valuable references for maize genetic improvement and variety breeding.

## Figures and Tables

**Table 1:** Statistical analysis of plant height (PH), ear height (EH), and the ratio of EH/PH (EH/PH) traits in 212 inbred lines of maize.

Traits	Environment	Mean ± SEM	Range	CV/%	Skewness	Kurtosis	H^2^/% ^a^
PH	21FY	152.68 ± 1.45	95.30–235.60	13.81	0.68	1.27	80.95
	21HN	168.11 ± 1.47	105.60–243.90	12.74	0.48	0.75	
	22FY	156.54 ± 1.43	112.80–226.50	13.30	0.55	0.26	
	22HN	183.66 ± 1.56	123.50–251.80	12.34	0.31	−0.09	
EH	21FY	50.96 ± 0.84	21.88–90.25	24.12	0.38	0.09	80.97
	21HN	54.72 ± 0.88	27.60–89.60	23.41	0.46	−0.30	
	22FY	52.06 ± 0.76	28.50–86.70	21.31	0.59	0.33	
	22HN	62.94 ± 0.81	42.10–99.00	18.77	0.52	−0.17	
EH/PH	21FY	0.33 ± 0.00	0.18–0.47	16.12	−0.15	0.06	79.87
	21HN	0.32 ± 0.00	0.20–0.48	16.45	0.21	0.01	
	22FY	0.33 ± 0.00	0.19–0.49	14.85	0.21	0.43	
	22HN	0.34 ± 0.00	0.24–0.51	13.40	0.63	0.92	

^a^ H^2^ represents the broad-sense heritability. SEM means standard error of mean. CV denotes the coefficient of variation.

**Table 2:** Stable SNP loci and the candidate genes identified in this study.

Traits	SNP	Chr	Position	*p* Value	PVE(%) ^a^	Enviroment	Candidate Gene	Gene Annotation
PH	AX-108020973	2	50,805,562	2.73 × 10^−10^	7.42	PH.22HN/ PH.BLUE	*GRMZM2G113629*	Protein disulfide isomerase-like
	AX-108022922	3	28,296,631	5.06 × 10^−10^	8.61	PH.21HN/ PH.22HN	*GRMZM2G375249*	Extensin-like protein
							*GRMZM2G076029*	Leucine-rich repeat extensin-like protein 3
							*GRMZM2G152689*	Phosphatidyl ethanol amine-binding protein
	AX-247241325	5	177,277,477	3.39 × 10^−17^	7.70	PH.22HN/ PH.BLUE	*GRMZM2G099989*	Exocyst complex component SEC6
							*GRMZM2G316366*	Encodes a MADS box protein
							*GRMZM2G492156*	MADS-box transcription factor
							*AC210598.3_FG003*	Phytochelatin synthase 1(PCS1)
EH	AX-108097244	8	135,948,728	2.68 × 10^−7^	38.49	EH.21FY/ EH.21HN/ EH.22HN/ EH.BLUE	*GRMZM2G464754*	WD-40 repeat family protein
							*AC207342.3_FG008*	DCD domain protein
							*GRMZM2G028733*	Ubiquitin-specific protease 7
EH/PH	AX-108096437	5	201,653,556	2.69 × 10^−9^	6.23	EH/PH.21FY/ EH/PH.BLUE	*GRMZM2G147377*	Lateral stability of cortical microtubules
							*GRMZM2G125432*	DNA binding transcription factors
							*GRMZM2G170595*	Histidinol phosphate aminotransferase 1

^a^ Proportion of the phenotypic variation explained by the SNP. Chr represents chromosomes. FY refers to Fengyang County, Anhui Province, while HN denotes Sanya City, Hainan Province.

## Data Availability

The data presented in this study are available in the article and Supplementary Materials.

## Supplementary Materials

The following supporting information can be downloaded at: https://www.mdpi.com/article/10.3390/plants14030481/s1, Table S1: the plant height (PH), ear height (EH), and EH/PH ratio of 212 maize inbred lines; Table S2: the information of 212 maize inbred lines in the panel and 26 representative inbred lines of various types; Table S3: summary of trait-associated SNPs, genetic loci, and candidate genes; Table S4: climate data for the maize cultivation periods in 2021 and 2022 are provided for Fengyang and Sanya.

## Abbreviations

Genome-wide association study (GWAS); plant height (PH); ear height (EH); phenotypic variation explained (PVE); quantitative trait locus (QTL); single nucleotide polymorphism (SNP); gibberellin (GA), brassinolide (BR), and strigolactone (SL); Improved Reid group (ImprReid); P group (P); Tang Si Ping Tou group (TSPT); Iodent group (IDT); Lancaster group (LAN); Early-maturing hard-grain cluster (EMHG); Lvda Red bone group (LRC); Reid group (Reid); X group (X); Mixed group (Mixed); W (Local glutinous group); T (Tropical group).
